# A machine learning model for predicting short-term in-hospital mortality in acute myocardial infarction with coexisting chronic obstructive pulmonary disease

**DOI:** 10.3389/fcvm.2026.1863785

**Published:** 2026-06-25

**Authors:** Weibin He, Jieli Sheng, Shuxiong Cai, Lihong Zheng, Zhenzhao Wang, Shujiao Zheng, Xinqi Lai, Chun Yang, Yiting Ke, Xiaohong Huang

**Affiliations:** 1 Department of Cardiology, Zhangzhou Affiliated Hospital of Fujian Medical University, Zhangzhou, Fujian, China; 2 Zhangzhou Health Vocational College, Zhangzhou, Fujian, China

**Keywords:** acute myocardial infarction, chronic obstructive pulmonary disease, machine learning, MIMIC-IV database, mortality, SHAP

## Abstract

**Background:**

Patients with acute myocardial infarction(AMI)complicated by chronic obstructive pulmonary disease (COPD) in the intensive care unit (ICU) face a significantly elevated risk of mortality. Therefore, timely and accurate risk stratification is critical for guiding clinical decision-making. However, validated interpretable prediction models for short-term mortality in patients with both AMI and COPD remain limited.

**Methods:**

We retrospectively extracted data from the MIMIC-IV database (version 3.1) and identified ICU patients with acute myocardial infarction complicated by chronic obstructive pulmonary disease. The primary outcome was 28-day in-hospital mortality. Five machine learning models were developed and compared: eXtreme Gradient Boosting (XGBoost), logistic regression (LR), gradient boosting decision tree (GBDT), light gradient boosting machine (LightGBM), and Adaptive Boosting (AdaBoost). Model discrimination was assessed using the area under the receiver operating characteristic curve (AUC), calibration was evaluated with calibration curves, and clinical utility was examined using decision curve analysis (DCA). In addition, SHAP (SHapley Additive exPlanations) analysis was used to provide interpretable visualization of model predictions.

**Results:**

A total of 662 ICU patients with acute myocardial infarction and chronic obstructive pulmonary disease were included, of whom 185 (27.9%) died within 28 days of hospital admission. Among the five machine learning models, the logistic regression (LR) model demonstrated superior discriminative performance, with an AUC of 0.782 in the validation cohort. The AUCs of the other models were 0.739 for XGBoost, 0.761 for LightGBM, 0.767 for GBDT, and 0.764 for AdaBoost. In addition, the LR model demonstrated good calibration and clinical utility. Eight variables were ultimately selected to build the prediction model, including age, heart rate (HR), respiratory rate (RR), lactate dehydrogenase (LDH), blood urea nitrogen (BUN), sepsis, *β*-blocker use (BB), and angiotensin-converting enzyme inhibitors/angiotensin receptor blockers (ACEI or ARB). SHAP analysis was subsequently applied to identify key predictors and enhance model interpretability.

**Conclusion:**

Using the logistic regression (LR), we developed a predictive model for 28-day in-hospital all-cause mortality in ICU patients with AMI and COPD. This model may help clinicians identify high-risk patients at an early stage, enabling more informed treatment decisions and more efficient allocation of medical resources.

## 1 Introduction

Acute myocardial infarction affects millions of individuals worldwide each year and remains a leading cause of death and disability (1, 2). Although advances in reperfusion therapy and secondary prevention strategies have markedly improved outcomes in patients with AMI, the presence of comorbidities continues to have a profound impact on clinical prognosis (3–6). Chronic obstructive pulmonary disease(COPD), a common respiratory disorder characterized by persistent airflow limitation, is one of the most frequent and clinically important comorbidities in patients with AMI (7, 8). Epidemiological studies indicate that the prevalence of COPD among patients with AMI ranges from 7% to 28%, with a substantial proportion of cases remaining newly diagnosed. Due to the large number of undiagnosed patients, the prevalence reported in registry-based studies is likely underestimated (9). For example, a meta-analysis including 6.4 million patients reported an overall COPD prevalence of 14.2% among individuals with coronary artery disease (8). In contrast, a study using objective pulmonary function screening identified a COPD prevalence as high as 18% among hospitalized AMI patients with a history of smoking (10).

COPD and AMI are linked through more than shared risk factors such as smoking (11). The two conditions interact via common pathophysiological mechanisms, including systemic inflammation and oxidative stress, creating a vicious cycle (12). COPD is fundamentally characterized by chronic inflammation of the airways and lung parenchyma; however, this inflammatory response is not confined to the lungs but spreads systemically through inflammatory mediators (13).

This systemic inflammation is reflected by persistently elevated circulating levels of pro-inflammatory mediators such as interleukin-6 (IL-6), C-reactive protein (CRP), and tumor necrosis factor-α (TNF-α) (14–16). Such a pro-inflammatory state can impair endothelial function and promote the formation and instability of atherosclerotic plaques (12, 17). Donaldson et al. reported that hospitalized patients with acute exacerbations of chronic obstructive pulmonary disease (AECOPD) have a significantly increased risk of AMI, especially within the first month after admission, with a 3- to 7-fold increase (18), and this elevated risk can persist for up to 1 year (19). The risk of myocardial infarction rises 2.27-fold within 1–5 days after an AECOPD episode (20). The U.S. EXACOS-CV study found that severe AECOPD was associated with a 30-day mortality hazard ratio (HR) of 5.09 (95% CI: 4.30–6.03), with cardiovascular event risk remaining elevated for 1 year (21). Dalal et al. showed that COPD patients with comorbid cardiovascular disease incur substantially higher healthcare costs compared with patients with COPD alone, including annual total and COPD-specific medical expenditures (22). Another study, using propensity score matching and weighted regression analyses, demonstrated that COPD increases the in-hospital mortality risk after percutaneous coronary intervention (PCI) in acute coronary syndrome (ACS) patients by 3.6–12.5 times (OR = 3.60–12.49). Therefore, accurate prediction of mortality in this patient population is critical for clinical decision-making and patient management (23).

This study aimed to develop predictive models for 28-day in-hospital all-cause mortality risk in patients with AMI complicated by chronic COPD using multiple machine learning algorithms. Models demonstrating superior predictive performance and clinical relevance were identified and selected. Furthermore, SHapley Additive exPlanations (SHAP) analysis was employed to interpret model outputs by identifying key clinical predictors.

## 2 Materials and methods

### 2.1 Data source and study population

The data for this study were derived from the MIMIC-IV database(version 3.1) (24). This publicly available, de-identified dataset contains comprehensive health records of patients admitted to the intensive care unit (ICU) of Beth Israel Deaconess Medical Center between 2008 and 2022. As all personal identifiers have been removed from the dataset, neither patient informed consent nor approval from an institutional review board or ethics committee was required. This study strictly adhered to the relevant guidelines for observational research outlined in the Strengthening the Reporting of Observational Studies in Epidemiology (STROBE) statement (25). Patients with acute myocardial infarction complicated by chronic obstructive pulmonary disease admitted to the ICU were identified using International Classification of Diseases, 9th and 10th Revision (ICD-9 and ICD-10) codes. The inclusion criteria were as follows: (1) age ≥18 years; (2) ICU length of stay of at least 24 h; and (3) first ICU admission. For patients with multiple ICU admissions, only the first admission was included. Patients with more than 30% missing values among candidate predictors were excluded before model development.

### 2.2 Data collection

Patients admitted to the ICU with a diagnosis of AMI complicated by COPD were enrolled, and the following data were collected: (1) demographic information: age, sex, height, and body weight; (2) vital signs: body temperature, heart rate, respiratory rate, and peripheral oxygen saturation (SpO₂); (3) laboratory parameters: complete blood count, liver and renal function, electrolytes, lactate, blood gas analysis, coagulation function, cardiac biomarkers, and NT-proBNP; (4) comorbidities: heart failure (HF), atrial fibrillation (AF), hypertension (HT), diabetes mellitus (DM), hyperlipidemia, pneumonia, chronic kidney disease (CKD), and sepsis; (5) medication-related variables; (6) interventions; and (7) outcome: 28-day in-hospital mortality.

All candidate predictors were extracted from data recorded within the first 24 h after ICU admission. For vital signs and laboratory parameters, values measured within the first 24 h after ICU admission were used. Medication-related variables were divided into prior home medication use and acute ICU medication use. Prior home medication use, including ACE inhibitors/angiotensin receptor blockers, beta-blockers, furosemide, and spironolactone, was defined as medication use documented before hospital admission or before ICU admission, rather than medications newly initiated during the subsequent ICU or hospital course. Acute ICU medications, including dopamine, dobutamine, epinephrine, norepinephrine, and phenylephrine, were considered only if administered within the first 24 h after ICU admission. Interventions, including mechanical ventilation, continuous renal replacement therapy (CRRT), percutaneous coronary intervention/percutaneous transluminal coronary angioplasty (PCI/PTCA), intra-aortic balloon pump (IABP), and coronary artery bypass grafting (CABG), were also considered only if initiated or performed within the first 24 h after ICU admission. Variables reflecting late ICU events, subsequent in-hospital complications, post-baseline treatments, or outcome-related information after the first 24 h were not included. The primary outcome was 28-day in-hospital all-cause mortality, defined as death occurring during hospitalization within 28 days after hospital admission.

### 2.3 Data preprocessing

Following data collection, the study cohort was first randomly divided into a training set and a validation set at a ratio of 7:3 before any preprocessing procedures were performed. All preprocessing procedures were fitted using the training set only and were subsequently applied to the validation set without refitting. Following data collection, rigorous data preprocessing was performed to ensure analytical accuracy and model stability. Data transformation was conducted, including the calculation of body mass index (BMI) from height and weight measurements. Outliers were handled using the 3× interquartile range (IQR) method to identify values outside the normal range, followed by further clinical review to confirm whether they constituted true outliers; confirmed outliers were replaced with the median value. Missing data were addressed with care: patient records and predictor variables with a missing rate exceeding 30% were removed, and remaining missing values were imputed using the K-nearest neighbor (KNN) algorithm. In addition, Z-score standardization was applied to transform all variables to a distribution with a mean of 0 and a standard deviation of 1, thereby eliminating differences in units of measurement.

### 2.4 Feature selection

After removing features with excessive missing values, a combination of multiple feature selection methods was applied for feature screening. Spearman correlation coefficients and variance inflation factor (VIF) values were calculated to identify features with strong correlations or multicollinearity; a Spearman correlation coefficient greater than 0.5 was indicative of strong inter-variable correlation, while a VIF value greater than 5 was indicative of multicollinearity among variables. The Boruta algorithm was subsequently applied for further feature selection, in which the importance of each feature was compared against that of randomly generated “shadow features” to determine whether it should be retained (26). Finally, recursive feature elimination (RFE) was employed to conduct a more refined importance evaluation of the remaining features.

### 2.5 Model construction, evaluation, and interpretation

Ten-fold cross-validation was performed within the training set for internal model assessment and model development, whereas the validation set was kept independent and used exclusively for final model evaluation. No hyperparameter tuning was performed; all models were implemented using predefined or default parameter settings. Five machine learning algorithms were used to develop predictive models, including eXtreme Gradient Boosting (XGBoost), Logistic Regression (LR), Gradient Boosting Decision Tree (GBDT), Light Gradient Boosting Machine (LightGBM), and AdaBoost Classifier (AdaBoost). Model predictive performance was evaluated on the validation set using the following metrics: area under the receiver operating characteristic curve (AUC), accuracy, sensitivity, specificity, positive predictive value (PPV), negative predictive value (NPV), and F1 score. The ROC curve was used to assess model discrimination, calibration curves to evaluate model calibration, decision curve analysis (DCA) to assess clinical utility, and the precision-recall (PR) curve to evaluate model performance under class imbalance. For the best-performing predictive model, the SHapley Additive exPlanations (SHAP) method was applied for result interpretation. Feature contribution plots, SHAP summary plots, and individual-level SHAP values were utilized to gain a comprehensive understanding of each feature's contribution to model predictions, to quantify the positive or negative influence of specific feature values on predictive outcomes, and to elucidate how these features collectively contributed to the final prediction output.

### 2.6 Statistical analysis

In the baseline description, normality testing of continuous variables was performed using the Shapiro–Wilk test. Normally distributed data were presented as mean ± standard deviation (SD), and between-group comparisons were performed using the t-test. Non-normally distributed data were expressed as median (interquartile range), and comparisons between groups were conducted using the Wilcoxon test. Categorical variables were presented as frequencies and percentages, and comparisons were made using the Chi-squared (*χ*^2^) test or Fisher's exact test. A two-sided *P* value < 0.05 was considered statistically significant. All statistical analyses were performed using R (version 4.2.3) and Python (version 3.11.4).

## 3 Results

### 3.1 Baseline characteristics

**Figure 1:**
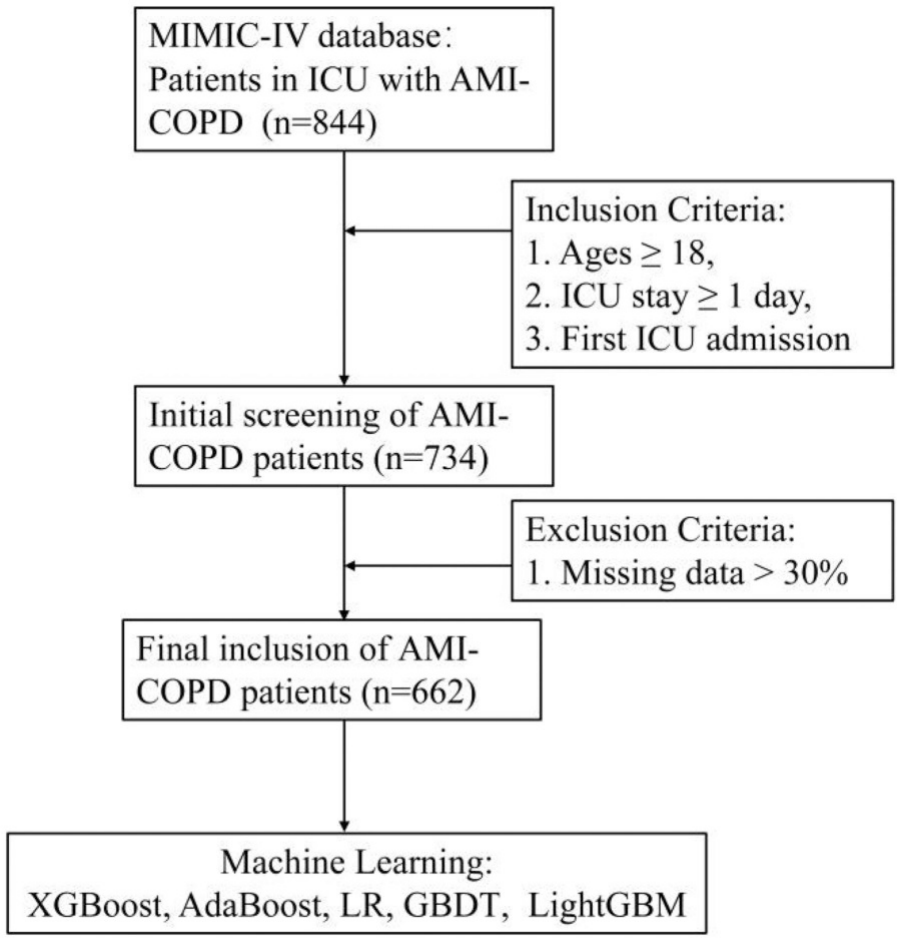
Patient selection flowchart. LR, logistic regression; GBDT, gradient boosting decision tree; AdaBoost, Adaptive Boosting; LightGBM, light gradient boosting machine; XGBoost, eXtreme Gradient Boosting.

A total of 844 patients with AMI complicated by COPD were initially extracted from the MIMIC-IV database; after applying the inclusion and exclusion criteria, 662 patients were ultimately enrolled. The screening process is detailed in Figure 1. Features with a missing rate exceeding 30% were excluded, including BMI, ALB, DDI, LDL, NEU, NT-proBNP, and TBIL (see Supplementary Figure S1). Baseline characteristics of the study population are summarized in Table 1, encompassing demographic data, vital signs, comorbidities, laboratory parameters, surgical procedures, mechanical ventilation, CRRT, and medication use. Among the 662 patients in the MIMIC-IV database, 185 AMI complicated by COPD patients died within 28 days of hospitalization, corresponding to a mortality rate of 27.9%. Compared with survivors, non-survivors exhibited significantly higher levels of Age, HR, APTT, Na, Lac, CKMB, RR, ALT, AST, BUN, Cr, and LDH, and significantly lower levels of PO₂, TCO₂, and pH (all *p* < 0.05). Significant differences were also observed between the survival and non-survival groups in the use of angiotensin-converting enzyme inhibitors/angiotensin receptor blockers (ACEI/ARB), long-acting beta-2 agonists (LABA), short-acting beta-2 agonists (SABA), beta-blockers (BB), spironolactone, dopamine, dobutamine, norepinephrine, and corticosteroids, as well as in the use of mechanical ventilation and CRRT (all *p* < 0.05). Furthermore, the proportion of patients with sepsis was significantly higher in the non-survival group compared with the survival group (*p* < 0.05), whereas no significant differences were observed between the two groups with respect to comorbidities including hypertension, diabetes mellitus, chronic kidney disease (CKD), atrial fibrillation, and heart failure.

**Table 1:** Baseline characteristics of AMI-COPD in the MIMIC-IV database.

Variables	Total (*n* = 662)	0 (*n* = 477)	1 (*n* = 185)	*P*
Demographics				
Age(years)	74.00 (68.00, 81.00)	73.00 (67.00, 79.00)	77.00 (70.00, 83.00)	**<**.**001**
Gender,n(%)				0.716
Female	265 (40.03)	193 (40.46)	72 (38.92)	
Male	397 (59.97)	284 (59.54)	113 (61.08)	
Vital signs				
Hr (bpm)	84.20 (74.17, 95.94)	82.42 (74.46, 92.75)	88.60 (73.00, 101.90)	**0**.**003**
RR(bpm)	19.79 (17.63, 22.18)	19.59 (17.42, 21.72)	20.48 (18.15, 23.38)	**<**.**001**
Spo2 (%)	96.43 (95.15, 98.00)	96.42 (95.36, 97.92)	96.52 (95.03, 98.18)	0.983
T ( °C)	36.80 (36.61, 36.98)	36.80 (36.63, 36.97)	36.80 (36.58, 37.01)	0.882
Laboratory indicators				
Wbc (K/uL)	12.20 (9.03, 17.00)	12.10 (8.80, 16.70)	12.60 (9.60, 17.60)	0.113
Rbc (m/uL)	3.38 (2.88, 3.95)	3.40 (2.89, 3.97)	3.33 (2.86, 3.93)	0.641
Plt (K/uL)	184.00 (137.00, 244.00)	187.00 (141.00, 246.00)	176.00 (128.00, 237.00)	0.095
Hb (g/dL)	10.00 (8.40, 11.60)	10.00 (8.50, 11.70)	10.00 (8.30, 11.30)	0.401
Hct (%)	31.10 (26.70, 36.70)	31.10 (26.90, 36.70)	31.00 (26.70, 36.40)	0.773
Na (mEq/L)	138.00 (135.00, 141.00)	138.00 (135.00, 140.00)	139.00 (135.00, 142.00)	**0**.**009**
K (mEq/L)	4.40 (4.00, 5.00)	4.40 (4.00, 4.90)	4.50 (4.10, 5.10)	0.160
Ca (mg/dL)	8.40 (7.90, 8.80)	8.40 (7.90, 8.80)	8.40 (8.00, 8.90)	0.435
Cl (mEq/L)	102.00 (98.00, 106.00)	103.00 (98.00, 106.00)	102.00 (97.00, 107.00)	0.992
Glu,(mg/dL)	135.00 (108.25, 180.00)	134.00 (108.00, 173.00)	143.00 (109.00, 198.00)	0.154
Ph (units)	7.35 (7.29, 7.40)	7.36 (7.30, 7.40)	7.33 (7.25, 7.39)	**0**.**001**
Pco2 (mmHg)	45.00 (39.00, 51.00)	44.00 (40.00, 50.00)	45.00 (38.00, 53.00)	0.912
Po2 (mmHg)	77.00 (43.00, 147.50)	81.00 (45.00, 176.00)	62.00 (39.00, 100.00)	**<**.**001**
Lac (mmol/L)	1.70 (1.30, 2.20)	1.60 (1.20, 2.20)	1.70 (1.40, 2.50)	**0**.**013**
Tco2 (mEq/L)	26.00 (23.00, 28.00)	26.00 (23.00, 28.00)	24.00 (21.00, 28.00)	**<**.**001**
Pt (sec)	13.80 (12.43, 15.90)	13.80 (12.40, 15.80)	13.80 (12.80, 16.70)	0.068
Aptt (sec)	31.60 (27.70, 36.38)	31.20 (27.40, 35.60)	31.60 (29.10, 38.60)	**0**.**008**
Inr	1.30 (1.10, 1.50)	1.30 (1.10, 1.50)	1.30 (1.20, 1.50)	0.058
Alt (IU/L)	24.00 (15.00, 34.00)	22.00 (15.00, 30.00)	26.00 (18.00, 41.00)	**<**.**001**
Ast (IU/L)	40.00 (27.00, 55.00)	37.00 (26.00, 47.00)	42.00 (31.00, 79.00)	**<**.**001**
Bun (mg/dL)	28.00 (18.00, 46.00)	25.00 (17.00, 41.00)	39.00 (24.00, 55.00)	**<**.**001**
Cr (mg/dL)	1.30 (0.90, 1.90)	1.30 (0.90, 1.80)	1.50 (1.20, 2.30)	**<**.**001**
Ldh (IU/L)	306.00 (252.00, 396.00)	289.00 (248.00, 361.00)	337.00 (274.00, 482.00)	**<**.**001**
Ckmb (ng/mL)	8.00 (4.00, 12.00)	8.00 (4.00, 11.00)	9.00 (5.00, 14.00)	**0**.**032**
Comorbidities				
HF, n(%)				0.801
NO	198 (29.91)	144 (30.19)	54 (29.19)	
YES	464 (70.09)	333 (69.81)	131 (70.81)	
AF, n(%)				0.587
NO	344 (51.96)	251 (52.62)	93 (50.27)	
YES	318 (48.04)	226 (47.38)	92 (49.73)	
HT, n(%)				0.581
NO	127 (19.18)	89 (18.66)	38 (20.54)	
YES	535 (80.82)	388 (81.34)	147 (79.46)	
DM, n(%)				0.052
NO	368 (55.59)	254 (53.25)	114 (61.62)	
YES	294 (44.41)	223 (46.75)	71 (38.38)	
Hyperlipidemia, n(%)				0.180
NO	291 (43.96)	202 (42.35)	89 (48.11)	
YES	371 (56.04)	275 (57.65)	96 (51.89)	
CKD, n(%)				0.318
NO	403 (60.88)	296 (62.05)	107 (57.84)	
YES	259 (39.12)	181 (37.95)	78 (42.16)	
Pneumonia, n(%)				0.112
NO	432 (65.26)	320 (67.09)	112 (60.54)	
YES	230 (34.74)	157 (32.91)	73 (39.46)	
Sepsis, n(%)				**<**.**001**
NO	221 (33.38)	193 (40.46)	28 (15.14)	
YES	441 (66.62)	284 (59.54)	157 (84.86)	
Drugs				
dobutamine, n(%)				**0**.**011**
NO	641 (96.83)	467 (97.90)	174 (94.05)	
YES	21 (3.17)	10 (2.10)	11 (5.95)	
Inputdopamine, n(%)				**0**.**005**
NO	640 (96.68)	467 (97.90)	173 (93.51)	
YES	22 (3.32)	10 (2.10)	12 (6.49)	
epinephrine, n(%)				0.730
NO	608 (91.84)	437 (91.61)	171 (92.43)	
YES	54 (8.16)	40 (8.39)	14 (7.57)	
norepinephrine, n(%)				**<**.**001**
NO	435 (65.71)	332 (69.60)	103 (55.68)	
YES	227 (34.29)	145 (30.40)	82 (44.32)	
glucocorticoids, n(%)				**<**.**001**
NO	480 (72.51)	363 (76.10)	117 (63.24)	
YES	182 (27.49)	114 (23.90)	68 (36.76)	
ACEI or ARB, n(%)				**<**.**001**
NO	484 (73.11)	313 (65.62)	171 (92.43)	
YES	178 (26.89)	164 (34.38)	14 (7.57)	
Bb, n(%)				**<**.**001**
NO	161 (24.32)	74 (15.51)	87 (47.03)	
YES	501 (75.68)	403 (84.49)	98 (52.97)	
Furosemide, n(%)				0.187
NO	149 (22.51)	101 (21.17)	48 (25.95)	
YES	513 (77.49)	376 (78.83)	137 (74.05)	
Spironolactone, n(%)				**0**.**046**
NO	630 (95.17)	449 (94.13)	181 (97.84)	
YES	32 (4.83)	28 (5.87)	4 (2.16)	
Laba, n(%)				**0**.**002**
NO	505 (76.28)	349 (73.17)	156 (84.32)	
YES	157 (23.72)	128 (26.83)	29 (15.68)	
Lama, n(%)				0.140
NO	538 (81.27)	381 (79.87)	157 (84.86)	
YES	124 (18.73)	96 (20.13)	28 (15.14)	
Sama, n(%)				0.890
NO	400 (60.42)	289 (60.59)	111 (60.00)	
YES	262 (39.58)	188 (39.41)	74 (40.00)	
Saba, n(%)				**<**.**001**
NO	346 (52.27)	226 (47.38)	120 (64.86)	
YES	316 (47.73)	251 (52.62)	65 (35.14)	
Other indicators				
Crrt, n(%)				**<**.**001**
NO	598 (90.33)	446 (93.50)	152 (82.16)	
YES	64 (9.67)	31 (6.50)	33 (17.84)	
PCI or PTCA, n(%)				1.000
NO	650 (98.19)	468 (98.11)	182 (98.38)	
YES	12 (1.81)	9 (1.89)	3 (1.62)	
Iabp, n(%)				0.373
NO	633 (95.62)	454 (95.18)	179 (96.76)	
YES	29 (4.38)	23 (4.82)	6 (3.24)	
Ventilation, n(%)				**0**.**013**
NO	60 (9.06)	35 (7.34)	25 (13.51)	
YES	602 (90.94)	442 (92.66)	160 (86.49)	

Hr, Heart rate; RR, Respiratory rate; Spo2, Peripheral oxygen saturation; T, Temperature; Wbc, White blood cell count; Rbc, Red blood cell count; Plt, Platelet count; Hb, Hemoglobin; Hct, Hematocrit; Na, Sodium; K, Potassium; Ca, Calcium; Cl, Chloride; Glu, Glucose; Ph, Hydrogen Ion Concentration; Pco2, Partial pressure of carbon dioxide; Po2, Partial pressure of oxygen; Lac, Lactate; Tco2, Total carbon dioxide; Pt, Prothrombin time; Aptt, Activated partial thromboplastin time; Inr, International normalized ratio; Alt, Alanine aminotransferase; Ast, Aspartate aminotransferase; Bun, Blood urea nitrogen; Cr, Creatinine; Ldh, Lactate dehydrogenase; Ckmb, Creatine kinase-MB; Hf, Heart failure; Af, Atrial fibrillation; Ht, Hypertension; Dm, Diabetes mellitus; Ckd, Chronic kidney disease;Crrt, Continuous renal replacement therapy; PCI or PTCA, Percutaneous coronary intervention or Percutaneous transluminal coronary angioplasty; Iabp, Intra-aortic balloon pump; ACEI or ARB, Angiotensin-converting enzyme inhibitor or Angiotensin receptor blocker; Bb, Beta-blocker; Laba, Long-acting beta agonist; Lama, Long-acting muscarinic antagonist; Sama, Short-acting muscarinic antagonist; Saba, Short-acting beta agonist.

Bold values indicate statistically significant P values, defined as *P* < 0.05.

### 3.2 Feature selection

**Figure 2:**
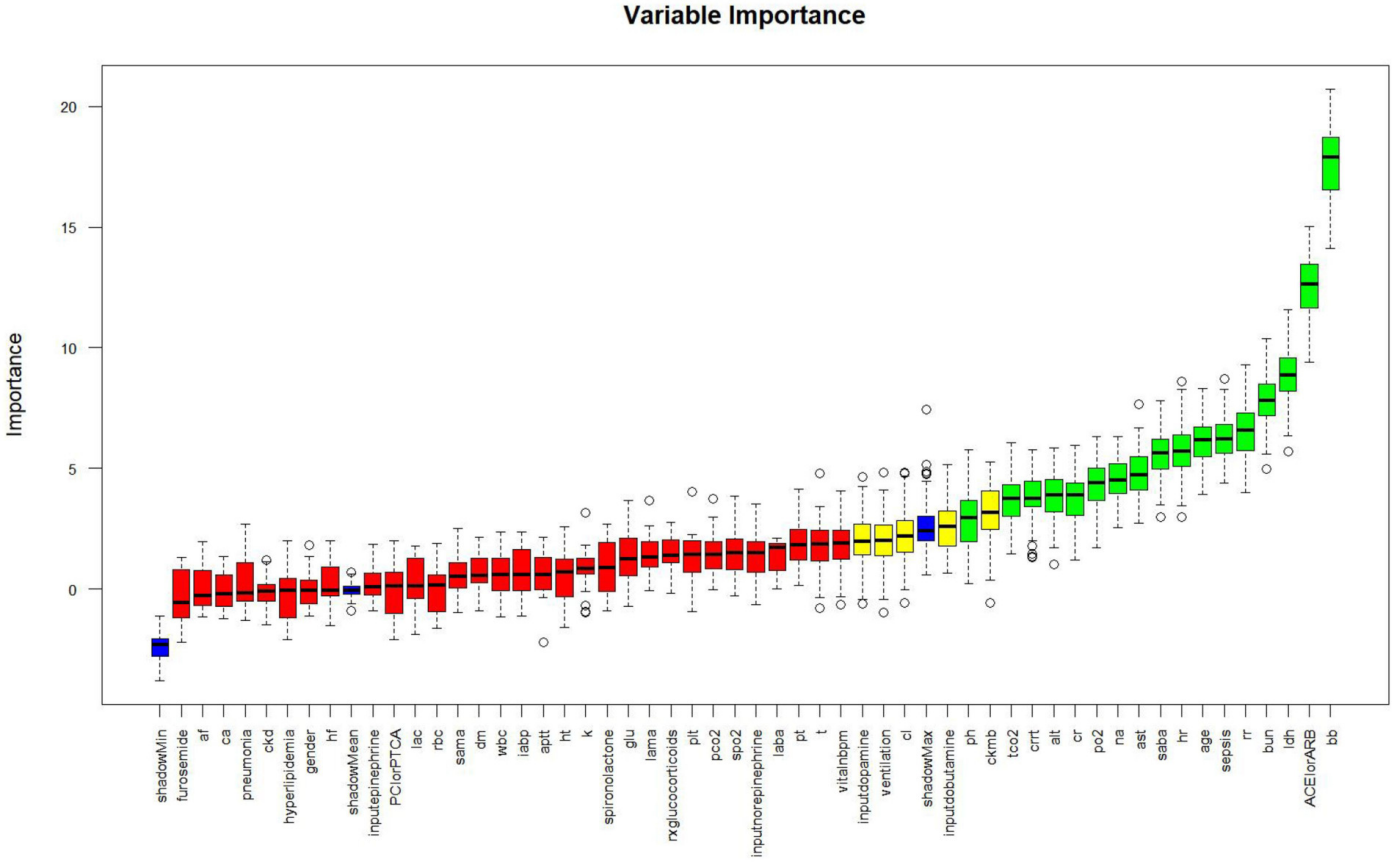
Feature selection analyzed by Boruta algorithm. The Boruta algorithm was applied during feature selection to compare the importance of original clinical variables with that of randomly generated shadow features. Green features indicate confirmed important predictors, red features indicate rejected unimportant predictors, yellow features indicate tentative predictors, and blue features represent shadow features. This procedure was performed as part of the feature selection workflow before final model construction.

Preliminary feature screening was performed based on a correlation heatmap and VIF values (see Supplementary Figures S2, S3), through which features with strong correlations or multicollinearity were excluded. Notably, these features also demonstrated apparent clinical relevance. Accordingly, HCT and Hb and INR were removed in the initial screening, while RBC and PT were retained. The Boruta algorithm was subsequently applied for further feature selection. Results of the Boruta method are typically presented in a visual format, in which different colors indicate the relative importance of each feature (see Figure 2). In the Boruta framework, blue represents shadow features, against which each original feature is compared to determine its importance. Red indicates features deemed unimportant, yellow denotes features with undetermined importance, and green indicates features of high importance. Finally, recursive feature elimination (RFE) was applied for further feature refinement. RFE operates by iteratively constructing models and progressively removing the features that contribute least to model performance, until the desired number of features is reached or no further improvement in model performance is observed. Ultimately, eight important features were identified: BB, ACEI/ARB, LDH, BUN, Sepsis, RR, Age and HR.

### 3.3 Construction and evaluation

**Figure 3:**
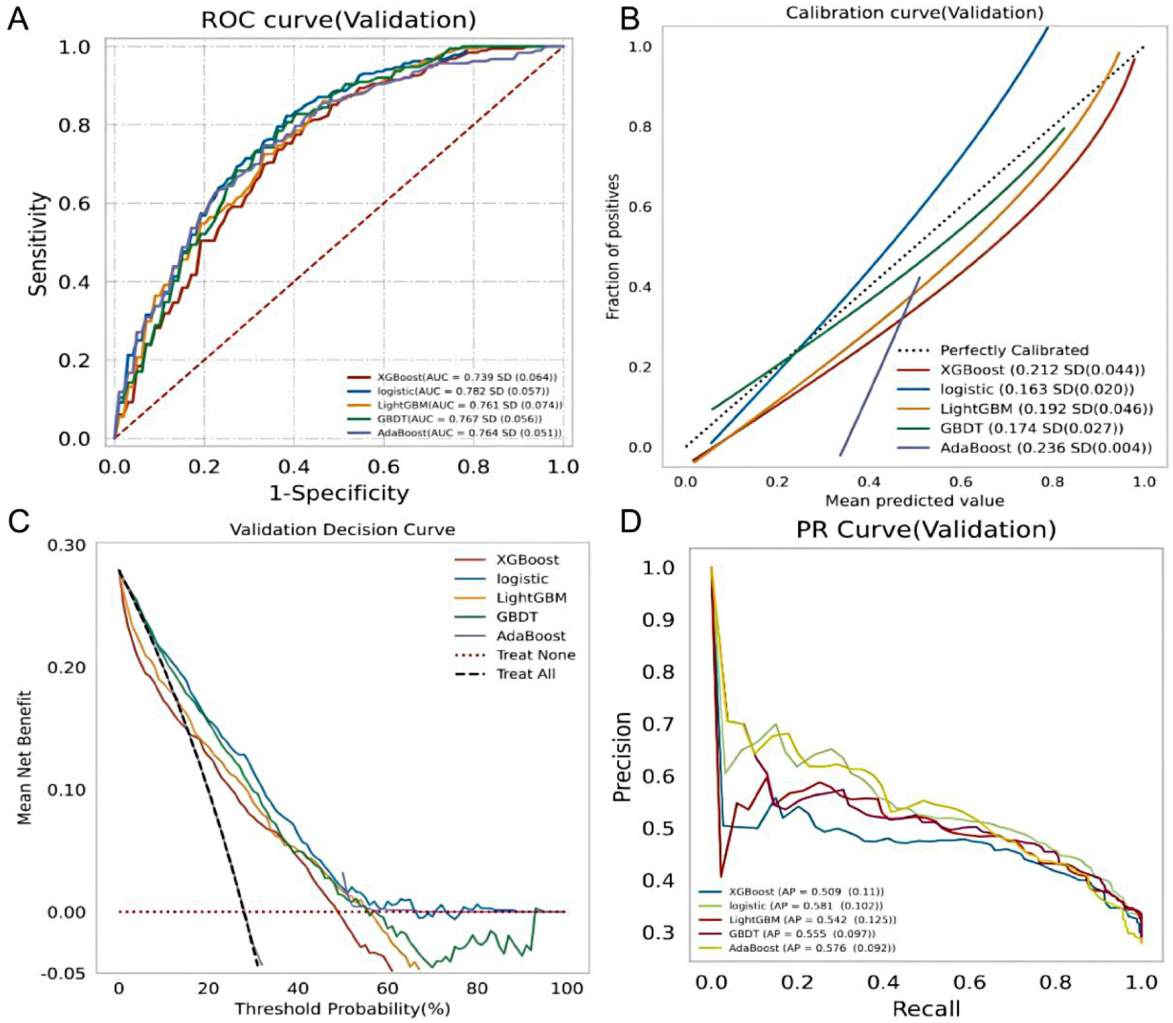
Model performance evaluation in the validation cohort. Performance of the five machine learning models, including logistic regression, XGBoost, LightGBM, GBDT, and AdaBoost, was evaluated in the validation cohort. **(A)** Receiver operating characteristic curves comparing model discrimination by AUC. **(B)** Calibration curves comparing predicted probabilities with observed 28-day in-hospital mortality. **(C)** Decision curve analysis evaluating the net clinical benefit of each model across different threshold probabilities. **(D)** Precision-recall curves assessing model performance under moderate class imbalance. AUC, area under the receiver operating characteristic curve; DCA, decision curve analysis; PR, precision-recall.

In this study, five machine learning algorithms were employed to construct predictive models: eXtreme Gradient Boosting (XGBoost), Logistic Regression (LR), Gradient Boosting Decision Tree (GBDT), Light Gradient Boosting Machine (LightGBM), and AdaBoost Classifier (AdaBoost). Model calibration was assessed graphically using calibration curves, which compared the predicted probabilities with the observed event rates across risk ranges. Decision curve analysis was further performed to evaluate the potential clinical utility of the models. The performance of these models is presented in Figure 3 and Table 2. The ROC curves (Figure 3A) demonstrated that the LR model achieved superior discrimination with an AUC of 0.782, while the remaining models performed as follows: XGBoost: 0.739, LightGBM: 0.761, GBDT: 0.767, and AdaBoost: 0.764. The calibration curve of the LR model closely followed the perfect calibration line (Figure 3B) and achieved a lower Brier score, indicating superior calibration. Based on the decision curve analysis (DCA) (Figure 3C), the LR model yielded the highest net benefit across a threshold probability range of 0%–85%, demonstrating superior clinical utility. The precision-recall (PR) curve (Figure 3D) showed that the LR model achieved the highest average precision (AP), attaining greater recall while maintaining high precision. Furthermore, the LR model demonstrated favorable performance across additional evaluation metrics, including accuracy (0.713), sensitivity (0.758), specificity (0.696), positive predictive value (0.492), negative predictive value (0.884), and F1 score (0.594). Accordingly, the LR algorithm was selected for construction of the final predictive model.

**Table 2:** Model performance comparison: AUC, accuracy, sensitivity, specificity, PPV, NPV, F1 score.

Models	AUC	Accuracy	Sensitivity	Specificity	PPV	NPV	F1 score
LR	0.782	0.713	0.758	0.696	0.492	0.884	0.594
GBDT	0.767	0.719	0.558	0.782	0.497	0.822	0.521
AdaBoost	0.764	0.715	0.671	0.732	0.495	0.853	0.567
LightGBM	0.761	0.731	0.260	0.914	0.521	0.762	0.342
XGBoost	0.739	0.724	0.222	0.918	0.498	0.753	0.305

LR, logistic regression; GBDT, gradient boosting decision tree; AdaBoost, Adaptive Boosting; LightGBM, light gradient boosting machine; XGBoost, eXtreme Gradient Boosting.

### 3.4 Model interpretation

**Figure 4:**
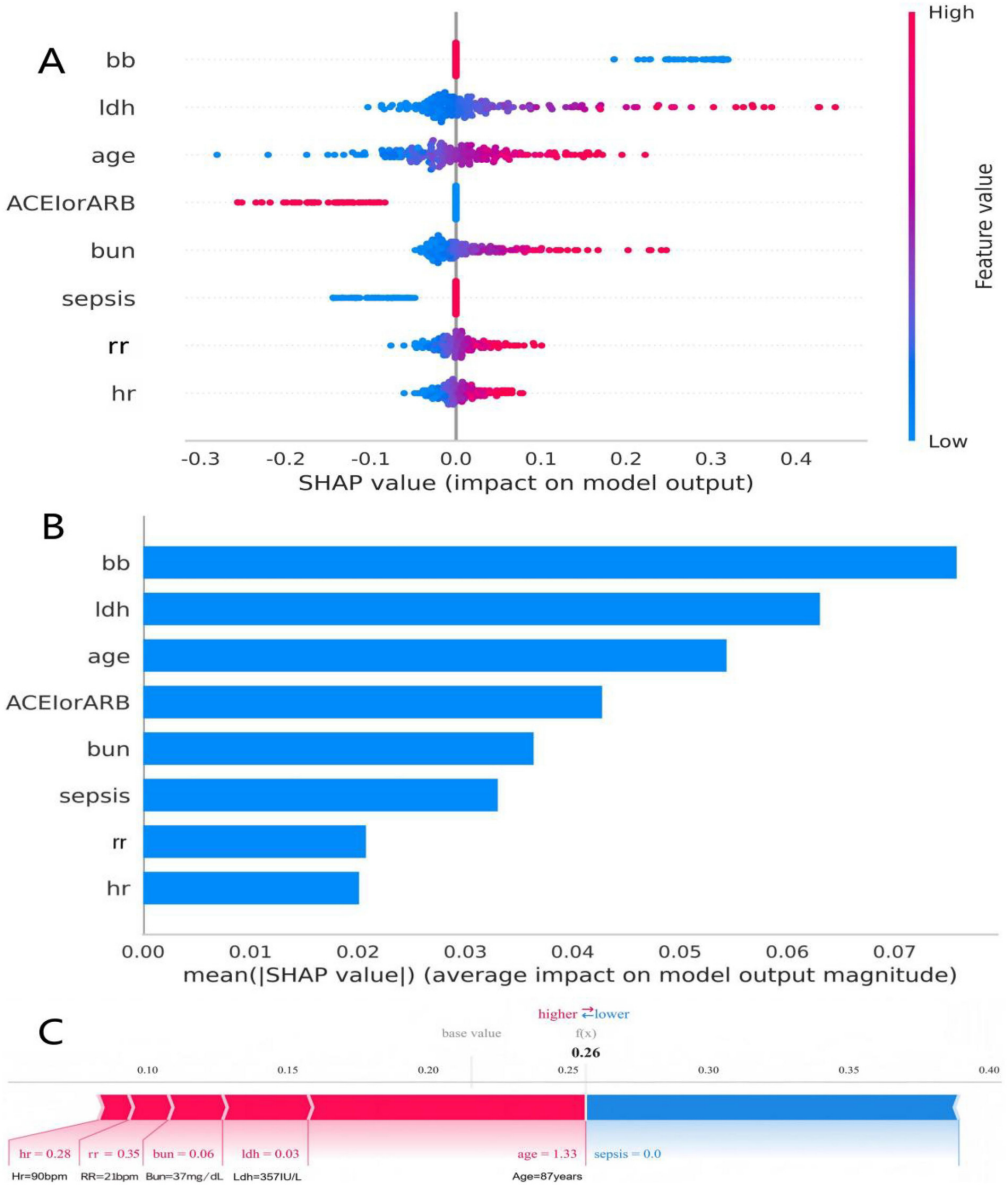
SHAP-based interpretation of the final logistic regression model. SHAP analysis was used to explain the contribution of each predictor to the final logistic regression model. **(A)** SHAP summary plot showing the distribution and direction of each feature's contribution to predicted 28-day in-hospital mortality. Each dot represents one patient, with red indicating higher feature values and blue indicating lower feature values. **(B)** Feature importance plot ranking predictors according to their mean absolute SHAP values. **(C)** Individual-level SHAP force plot illustrating how specific clinical features increased or decreased the predicted mortality risk for a representative patient. SHAP, SHapley Additive exPlanations.

The SHAP method was employed to explain model predictions. Global interpretability describes the overall influence of all features included in the model and the relationships among them, while local interpretability describes the specific contribution of each feature to the predicted outcome for an individual patient. Global interpretability was visualized using the SHAP Summary Plot, Feature Contribution Plot, and Dependence Plots, while local interpretability was visualized using the SHAP Force Plot. In the SHAP Summary Plot (Figure 4A), the *y*-axis represents the features and the *x*-axis represents the magnitude of each feature's impact on the predicted outcome. Each dot represents a single sample, with red dots indicating high feature values and blue dots indicating low feature values. As illustrated in Figure 4A, higher values of LDH, age, BUN, heart rate (HR), and respiratory rate (RR) (red dots) were associated with greater predicted mortality risk. Conversely, patients without sepsis (blue dots) were associated with lower predicted risk, while patients not receiving beta-blockers (BB) were associated with increased predicted risk, and those receiving ACEI/ARB were associated with reduced predicted risk. The Feature Contribution Plot (Figure 4B) reveals that the predictive importance of the eight included features ranked in descending order as follows: BB, LDH, age, ACEI/ARB, BUN, sepsis, RR, and HR.

The SHAP Force Plot offers an instance-level visualization of each feature's contribution to the model's predicted outcome. In the SHAP Force Plot, each feature's contribution is represented by a force arrow, where blue arrows indicate a negative influence on the prediction (pointing left, reducing the predicted value) and red arrows indicate a positive influence (pointing right, increasing the predicted value). As illustrated in Figure 4C, Age = 87 years, RR = 21 bpm, HR = 90 bpm, LDH = 357 IU/L, and BUN = 37 mg/dL each independently increased the predicted risk of 28-day in-hospital mortality, whereas the absence of sepsis decreased the predicted risk of 28-day in-hospital mortality.

## 4 Discussion

In this study, we developed and internally validated a short-term mortality risk prediction model for ICU patients with acute myocardial infarction complicated by chronic obstructive pulmonary disease. Among five machine learning algorithms, the logistic regression model demonstrated the most favorable overall performance, with acceptable discrimination, graphical calibration, interpretability, and clinical applicability. Although some complex ensemble models showed high apparent discriminative performance, their clinical usefulness should be interpreted cautiously, particularly when sensitivity is limited in the validation set. For a mortality prediction tool, model selection should not rely solely on AUC, but should also consider sensitivity, calibration, decision-curve analysis, and interpretability.

To our knowledge, this is among the first studies to construct a machine learning-based short-term mortality prediction model specifically for ICU patients with AMI-COPD comorbidity. Previous prediction studies have mainly focused on either AMI or COPD as single-disease populations, whereas integrated mortality prediction models for patients with both conditions remain limited (27–34). This gap is clinically relevant because COPD may modify the prognosis of AMI patients, and conventional AMI risk scores may underestimate mortality risk in patients with coexisting COPD (32). In the present study, the final logistic regression model incorporated eight readily available clinical variables, including age, heart rate, respiratory rate, lactate dehydrogenase, blood urea nitrogen, sepsis, *β*-blockers, and angiotensin-converting enzyme inhibitors/angiotensin receptor blockers. These variables were restricted to the first 24 h after ICU admission, supporting the potential value of the model for early risk stratification.

Logistic regression has been widely used in clinical prognostic modeling because of its simplicity, interpretability, and computational efficiency (35–37). Compared with complex ensemble algorithms, logistic regression is less prone to overfitting when the number of predictors is modest and the event number is limited. Its transparent model structure also facilitates clinical understanding and implementation. Therefore, in this specific setting, logistic regression may provide a more balanced option than more complex algorithms for early mortality risk prediction.

The predictors identified in the final model are clinically plausible. LDH and BUN have been associated with adverse outcomes in AMI or COPD populations and may reflect myocardial injury, tissue hypoxia, renal dysfunction, impaired perfusion, neurohormonal activation, and systemic illness burden (38–44). Sepsis is closely associated with organ dysfunction and high mortality in critically ill patients (45). Advanced age reflects reduced physiological reserve, immunosenescence, chronic inflammation, and a higher burden of comorbidities, all of which may contribute to poor prognosis in AMI and COPD populations (46–48). Elevated heart rate and respiratory rate may indicate sympathetic activation, hemodynamic instability, hypoxemia, metabolic acidosis, or respiratory compensation, and previous studies have shown that these parameters are associated with adverse outcomes in cardiovascular and pulmonary diseases (49–55). These routinely available variables may help clinicians rapidly identify patients at increased risk of early deterioration.

Medication-related predictors, including *β*-blockers and ACEI/ARB, should be interpreted with caution. Although these variables contributed to model prediction, their associations should not be regarded as causal effects. In critically ill patients, medication use may reflect clinical stability, treatment eligibility, physician decision-making, and contraindications. Patients who were able to receive *β*-blockers or ACEI/ARB may have had better baseline hemodynamic status or fewer contraindications, which could partially explain their lower predicted risk. Previous evidence suggests that cardioselective *β*-blockers may be safe in patients with AMI-COPD, and ACEI/ARB may provide cardiovascular benefit in patients with COPD and coronary artery disease (56, 57). Nevertheless, the present findings should be interpreted as predictive associations rather than evidence of treatment efficacy.

The present study has several clinical implications. First, it focuses on a high-risk comorbid population that is often underrepresented in disease-specific prediction studies. Second, the selected predictors are routinely available during the early ICU period, which supports potential clinical implementation. Third, SHAP-based interpretation improves model transparency by showing the contribution of each predictor at both the population and individual patient levels. This may help clinicians understand the model output and identify major contributors to individual risk. However, the model should be viewed as a risk stratification aid rather than a substitute for clinical judgment.

This study has several limitations. First, this was a retrospective study based on a single-center critical care database, and the lack of external validation may limit the generalizability of the findings. Multicenter prospective studies with larger sample sizes are needed to validate the proposed model. Second, although candidate predictors were restricted to variables recorded within the first 24 h after ICU admission, some variables, particularly medication use and sepsis, may still reflect early clinical decisions or evolving disease processes rather than purely baseline characteristics. Third, although variables and patient records with more than 30% missingness were excluded, some retained variables still required K-nearest neighbor imputation, which may have introduced uncertainty into model estimates. Fourth, the outcome event rate suggested moderate class imbalance, and some models showed limited sensitivity despite favorable AUC values. Future studies should explore threshold optimization, cost-sensitive learning, resampling strategies, and prospective validation to improve clinical sensitivity. Fifth, important clinical information, such as echocardiographic parameters, coronary angiographic characteristics, pulmonary function data, and detailed treatment strategies, was not fully available in the database. Finally, this study was designed for prediction rather than causal inference.

## 5 Conclusion

We developed an interpretable short-term mortality prediction model for ICU patients with AMI complicated by COPD. Logistic regression demonstrated the most favorable balance of discrimination, calibration, interpretability, and clinical applicability. The model incorporated eight routinely available clinical variables and was further interpreted using SHAP analysis. These findings suggest that an interpretable prediction model may assist early risk stratification in this high-risk comorbid population, although external validation and prospective evaluation are required before clinical implementation.

## Data Availability

The datasets presented in this study can be found in online repositories. The names of the repository/repositories and accession number(s) can be found in the article/Supplementary Material.
