# Supplementary figures and images for: A machine learning model for predicting short-term in-hospital mortality in acute myocardial infarction with coexisting chronic obstructive pulmonary disease
Source: Front Cardiovasc Med. 2026 Jun 25;13:1863785. doi: 10.3389/fcvm.2026.1863785 (PMC13346180; doi:10.3389/fcvm.2026.1863785)

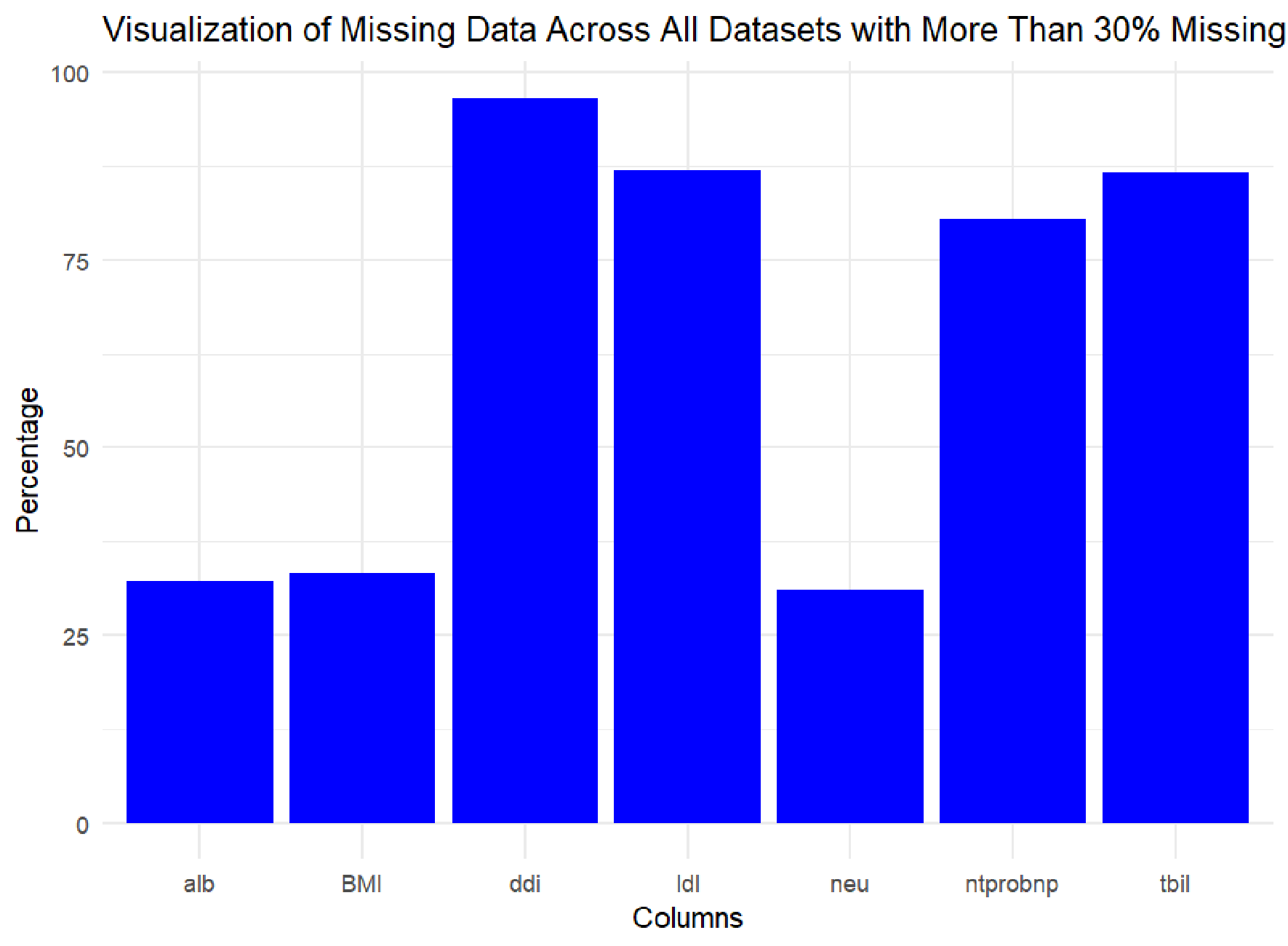

Supplement: Supplementary file 1 [file image1.tiff]

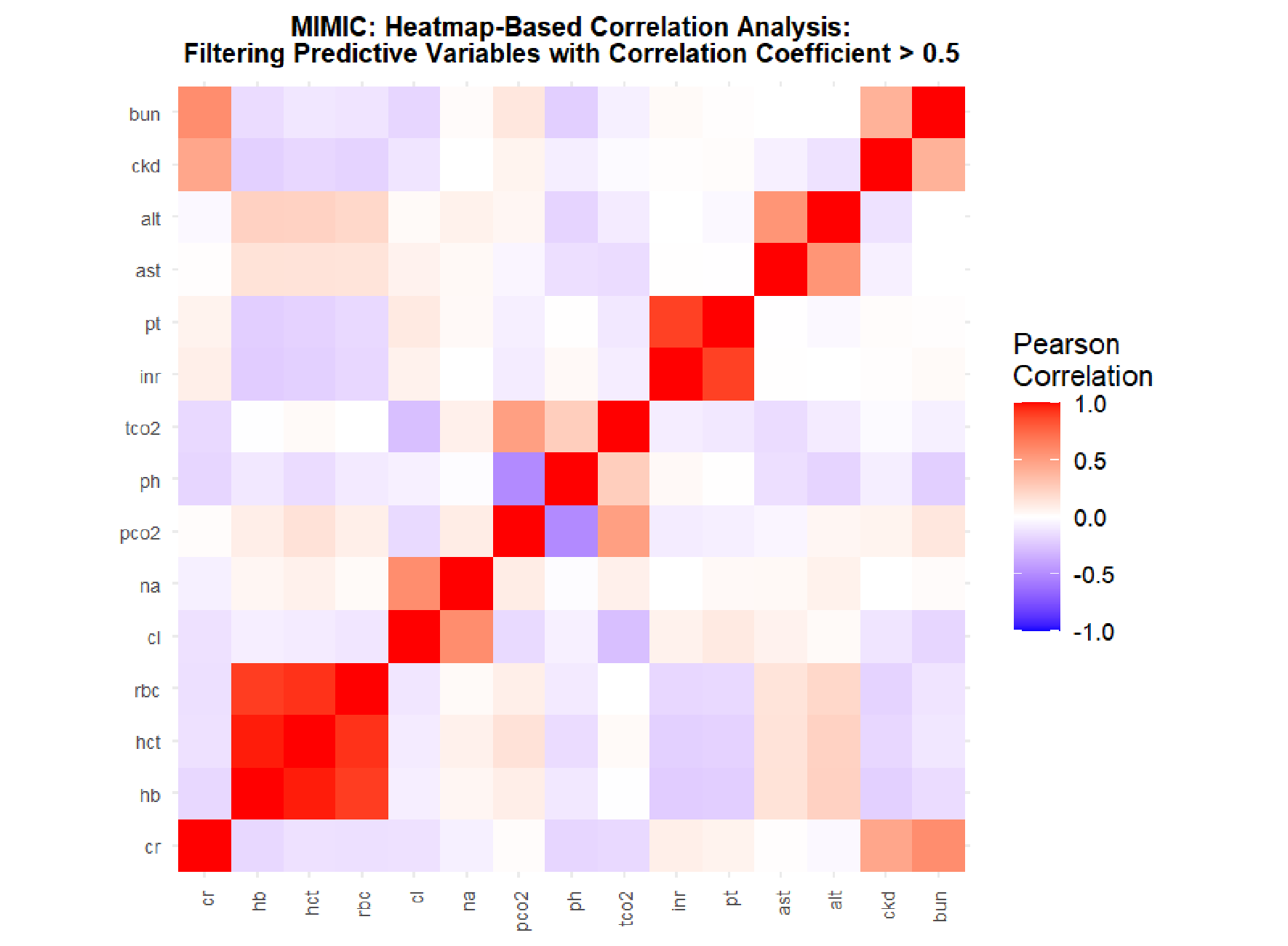

Supplement: Supplementary file 2 [file image2.tiff]

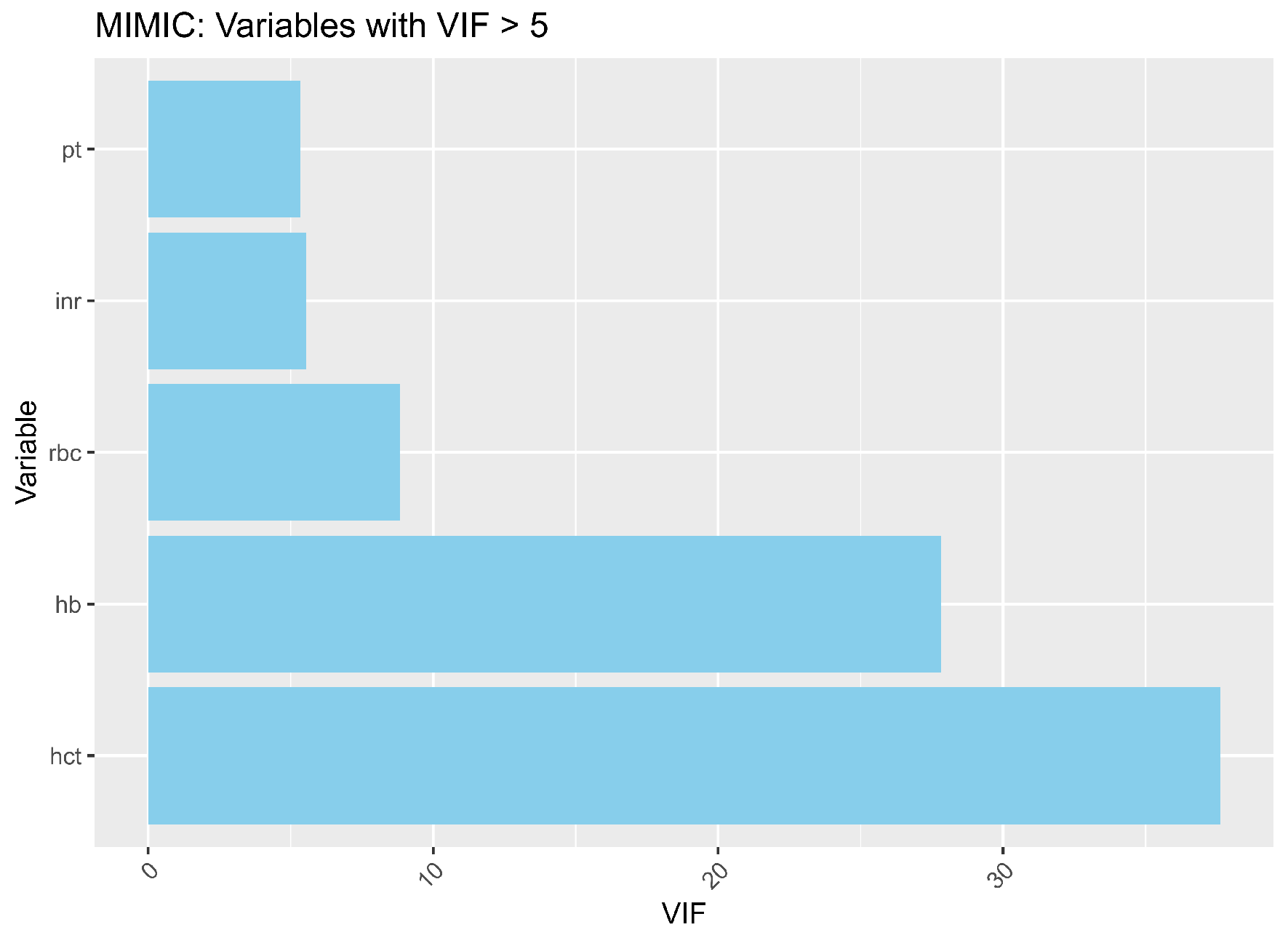

Supplement: Supplementary file 3 [file image3.tiff]
